# Optimizing Varicella Vaccination Strategy: A Study on Age and Dose Impacts on Antibody Levels

**DOI:** 10.3390/vaccines13010023

**Published:** 2024-12-30

**Authors:** Qing He, Yang Xu, Yilan Li, Pinting Zhu, Lei Luo

**Affiliations:** 1 Guangzhou Center for Disease Control and Prevention, Guangzhou 510440, China; heqingnzzz@foxmail.com (Q.H.); gzcdc_xuy@gz.gov.cn (Y.X.); gzcdc_liyilan@gz.gov.cn (Y.L.); 2 Institute of Public Health, Guangzhou Medical University and Guangzhou Center for Disease Control and Prevention, Guangzhou 511436, China

**Keywords:** varicella, seroepidemiology, vaccination

## Abstract

Seropositivity study of Varicella in Healthy Populations in Guangzhou, China. Infection with varicella-zoster virus (VZV) leads to skin and mucous membranes blisters and the complications can be life threatening. A seroepidemiological study conducted from 2020 to 2022 in Guangzhou, China, aimed to evaluate varicella antibody levels. We measured varicella antibody concentrations using an enzyme-linked immunosorbent assay. A total of 3300 people were enrolled in the study. The mean varicella antibody level was 171.2 mIU/mL (95% CI: 158.9, 184.4), with an overall positivity rate of 67.00% (95% CI: 65.37, 68.60). The mean level of those positive subjective was 581.2 mIU/mL (95% CI: 552.3, 611.5). Varicella antibody levels were found to be influenced by age, vaccination dosage, and history of varicella infection. Antibody level increased with age and the number of vaccinations. The antibody induced by the varicella vaccine remained at protective levels for at least 6 years post-vaccination. We recommend two doses of the varicella vaccine for both children and adults and the integration of the varicella vaccine into the national routine immunization program.

## 1. Introduction

The varicella-zoster virus (VZV),which belongs to the α-herpesvirus subfamily, is a double-stranded DNA virus with a single serotype [1]. VZV is transmitted from person to person through inhalation of respiratory droplets from patients and direct contact with infected secretions [2]. In temperate climates where vaccination is not practiced, infection with VZV approaches 100% by the age of forty [2]. After infection, immunity to these diseases appears to persist for life and second attacks are rare. Infection with VZV leads to two different clinical manifestation: varicella (also known as chickenpox) and zoster (also known as shingles) [3]. Primary infection with VZV leads to varicella. During primary infection, VZV hijacks T cells to disseminate to the skin and establishes latency in ganglia, while reactivation from a latent state in neurons results in zoster [4]. Both varicella and zoster are characterized by viral replication in the epidermis of the skin and mucous membranes, leading to the formation of blisters [3]. The most common complications in children are secondary bacterial infections caused by skin blisters, such as Streptococcus [5,6]. A variety of extracutaneous complications can also occur, including pneumonia, encephalitis, cerebellar ataxia, arthritis, hepatitis, pericarditis, and orchitis [7]. Secondary infection with Group A Streptococcus following varicella can be life threatening [5,8]. Infection with the varicella-zoster virus during pregnancy can endanger the fetus and newborn [7].

Vaccines are the most effective way of preventing varicella [2]. The VZV vaccine contains live-attenuated VZV virus, which induces a specific immune response in the body. Currently, the VZV vaccine strains include the Oka strain and MAV/06 strain. The Oka strain was the first attenuated live virus to be widely utilized in varicella vaccines [9]. Except for the varicella vaccine using MAV/06 strain licensed in South Korea, all VZV vaccines available worldwide are based on the Oka strain [2].

Before 1995, there was no varicella vaccine available in Guangzhou, China, and no effective drugs for prevention or treatment, so the disease was endemic. Varicella was designated as a local compulsory notifiable infectious disease in Guangzhou after 1995, managed as a Class C infectious disease, requiring cases to be reported within 24 h of diagnosis [10]. Simple measures should be taken to prevent the disease, such as home quarantine for patients in school or kindergarten until no new lesions (macules, papules, etc.) appear on the skin and mucous membranes within 24 h, and health monitoring for 2 weeks for their close contacts, etc. [11].

The highly contagious nature of varicella in schools prompted the government to implement control measures in 2012: emergency vaccination for susceptible children once a varicella outbreak was identified in a school [12]. Even after emergency vaccination for each outbreak, the annual number of varicella outbreaks in schools remained stubbornly high. Varicella outbreaks had the highest occurrence rate compared to other infectious disease in school [11]. Guangdong province introduced a two-dose immunization program for children to prevent the disease in 2017 [13]. Children aged 12–24 months are recommended to receive the first dose of the varicella vaccine, and children aged 4–6 years are recommended to receive the second dose. Until now, the varicella vaccine has not been included in Guangzhou’s immunization program; the vaccines are paid for by the vaccinee or their guardian and not mandatory for children.

Since the implementation of the two-dose varicella immunization program in November 2017, the incidence of varicella has dropped dramatically in Guangzhou, from 818.7/100,000 per year (2014–2019) to 251.12/100,000 per year (2020–2023). The high-risk group shift from children aged 1–14 to children under 1 year old [14]. The incidence rate has declined across the entire population. The annua incidence rate among the 0-8-year-old population decrease sharply from 1024.7/100,000 per year and 452.7/100,000 per year to 177.6/100,000 per year, for 2014–2018, 2019–2021, and 2022–2023, respectively. Meanwhile, the incidence rate among adults showed a small decline from 166.3/100,000 per year and 141.2/100,000 per year to 34.5/100,000 per year, for 2014–2018, 2019–2021, and 2022–2023. A significant reduction was particularly evident among the kindergarten and primary school populations. The number of reported varicella outbreaks in school dropped from 14.5 to 4.25 per year [10].

Antibody levels against infectious diseases, ascertained through serosurveillance, are influenced by vaccination and historical exposure to infection, serving as a biomarker for individual immune competence against specific pathogens and a metric for assessing the efficacy of vaccines within the immunized population. Surveillance of immunity levels against vaccine-preventable diseases is a mandated duty of the Centers for Disease Control and Prevention, as prescribed by the Vaccine Administration Law of the People’s Republic of China. An annual seroepidemiological assessment is conducted to evaluate the humoral immune response to vaccine-preventable communicable diseases among the healthy population in Guangzhou, China.

The purpose of this study is to evaluate the varicella antibody levels in the entire population and in different age groups in Guangzhou, analyze the potential factors that may affect the population’s antibody levels, and assess the impact of vaccination status and viral infection on antibody levels.

## 2. Materials and Methods

### 2.1. Study Design

Annual monitoring of population immunity levels in healthy individuals within communities was conducted in Guangzhou. One or more communities from each district were selected using simple random sampling. Based on the calculation formula for a cross-sectional study, n = *u_α/_*_2_^2^*p*(1 − *p*)/*δ*^2^, the minimum number of subjects required was determined to be 1155, with an expected varicella antibody seroprevalence of 50%, a precision of 0.05, an allowable error of 0.05, and a design effect of 3. There are 11 districts in Guangzhou, with an average of approximately 100 subjects per district. Each district recruited at least 8 subjects in each age group (0–5 months, 6–11 months, and 1, 2, 3, 4 years); at least 6 subjects in the age groups 5, 6, 7, 8, 9, 10–19, and 20–29 years; and at least 5 subjects in the age groups 30–59 and ≥60 years.

Participants’ venous blood, demographic information (age, sex, address, household registration, date of birth, and date of sampling), and history of varicella were collected by community staff from July to December.

Varicella vaccine vaccination history (doses, vaccination dates) was collected from the Guangdong Vaccine Circulation and Vaccination Management Information System by matching participants’ names, sexes, dates of birth, and addresses. The Vaccination Management Information System has been operational since 1997. Considering the year the information system was developed and potential subjects’ recall bias, we only collected vaccination information for children under 19 years old from the Information System.

Inclusion criteria: (1) participants must have a resting axillary temperature measured at the armpit using a standard clinical mercury thermometer. The temperature must be recorded as 37.0 °C or lower. (2) Participants must be aged between 0 and 79 years old at the time of enrollment. (3) Participants must have resided in Guangzhou for more than 3 years at the time of enrollment. (4) Participants aged 18 years or older must have normal blood pressure and heart rate (systolic blood pressure: 90–139 mmHg, diastolic blood pressure: 60–89 mmHg; heart rate: 60–100 beats per minute). If the initial measurement of blood pressure and heart rate is abnormal, it is recommended to rest quietly for at least 5 min before taking a subsequent measurement. Blood pressure measurements are not taken for individuals under the age of 18. (5) Participants must demonstrate a clear understanding and willingness to undergo the required blood sampling procedure and complete a brief questionnaire survey.

Exclusion criteria: (1) individuals’ axillary temperature ≥ 37.1 °C. (2) Individuals with immunodeficiency, post-organ transplant, or on hemodialysis are excluded. (3) Individuals who have participated in or are currently participating in other clinical trials within three months prior to screening are excluded. (4) Individuals who have received corticosteroids within 6 weeks. (5) Individuals who have received immunoglobulin or other blood products within 3 months. (6) Individuals who had impaired immune function or immunodeficiency.

All study participants gave written informed consent before the initiation of the study.

### 2.2. Serologic Evaluations

Blood samples were centrifuged at 3500× *g* for 15 min and sera were transferred into 2 mL cryotubes under sterile conditions. Serum samples were stored at −80 °C at the Guangzhou Center for Disease Control and Prevention (GZCDC). Before testing, sera were allowed to stand for 1 h and processed when they reached room temperature. An enzyme-linked immunosorbent assay (SERION ELISA classic Varicella Zoster Virus IgG; Virion/Serion, Würzburg, Germany) was used to quantitatively measure varicella IgG titers. The assay was performed by GZCDC from February to July each year, according to the manufacturer’s instructions, and optical density was measured at wavelengths of 405 and 620 nm using a spectrophotometer (Tecan, Sunrise, Männedorf, Austria). Results were expressed in milli-International Units (mIU/mL).

According to the manufacturer’s instructions, antibodies against varicella were defined as <50 mIU/mL as negative, ≥50 mIU/mL and <100 mIU/mL as equivocal, and ≥100 mIU/mL as positive. To facilitate the analysis, seroprotection was defined with a cut-off value of 100 mIU/mL for antibodies against varicella in this study.

### 2.3. Statistical Analysis

Data processing and analysis were performed using R version 4.3.0, along with the Storm Statistical Platform (www.medsta.cn/software, accessed on 7 August 2024). The varicella antibody concentration was recorded as geometric mean concentrations (GMCs) and analyzed in logarithmic form. Two-sample *t*-tests (or Wilcoxon two-sample tests), one-way ANOVA (or Kruskal–Wallis tests), or χ^2^ tests were used to compare the differences in varicella antibody concentration or demographic information between groups within the same doses. Mann–Kendall’s test was used to test the trend of antibodies by age group and varicella vaccine doses. Multivariate regression analysis with a stepwise method examined the association between log-transformed varicella antibody concentration and various groups.

### 2.4. Ethical Considerations

The Ethics Committee of the Guangzhou Center for Disease Control and Prevention reviewed and approved the study protocol (identification code: GZCDC-ECHR-2023P0037). Participants or their guardians were informed that their participation was voluntary.

## 3. Results

A total of 3300 subjects were enrolled from 2020 to 2022 in the study, ranging in age from newborn to 79 years old, with a median age of 5 and an average age of 10.37 years. The ratio of male to female subjects was 1.03:1. The ratio of local residents to migrant residents was 3.72:1. Over 80% (2712/3300) of the subjects were aged between 0 and 14 years, with 93.22% (2528/2712) having definite varicella vaccine dose records and vaccination dates. Additionally, 2.3% of the subjects had a history of varicella.

### Varicella Antibody GMCs and Positive Rates

The overall varicella IgG-specific antibody GMCs were 171.2 mIU/mL (95% CI: 158.9, 184.4) and the mean level of those positive subjects were 581.2 mIU/mL (95% CI: 552.3, 611.5). The overall positive rate of varicella antibodies was 67.0% (95% CI: 65.4, 68.6).

There was a statistically significant increase in GMCs and positive rate with age, from 56.2 mIU/mL at 0 years to 610.5 mIU/mL at 50 years and above, from 42.5% in the age group 0 years to 96.0% in the age group 51 years and above (*p* < 0.05). Varicella IgG-specific antibody levels showed a statistically significant increase in subjects who received varicella vaccinations, from 0 doses (46.5 mIU/mL) to two doses (340.2 mIU/mL). There were significant differences in GMCs and positive rate among different groups for subjects who received one dose (*p* < 0.05) or two doses (*p* < 0.001). The varicella antibody levels showed a statistically significant increase with the number of doses of the varicella vaccine received, from 46.5 mIU/mL with 0 dose to 342.2 mIU/mL with two doses for GMCs, from 40.7% with 0 doses to 83.4% with two doses for a positive rate (Table 1).

Females exhibited higher varicella IgG-specific antibody GMCs and positive rates than males. The varicella IgG-specific antibody GMCs and positive rates for women of childbearing age were 443.2 mIU/mL and 87.3%, which were significantly higher than those of other women. Local residents had higher varicella IgG-specific antibody GMCs and positive rates than non-local residents. The central urban area had a lower varicella antibody GMCs and positive rate compared to the suburban and outer suburban areas. Subjects with a history of VZV infection exhibited higher antibody GMCs and positive rates compared to those without such a history. Subjects born before 1995 had the highest antibody GMCs and positive rates compared to others (Table 1).

Differences in varicella IgG-specific antibody GMCs and positive rates among groups defined by age, household, sex, area, and vaccine doses were all statistically significant (*p* < 0.001).

**Figure 1:**
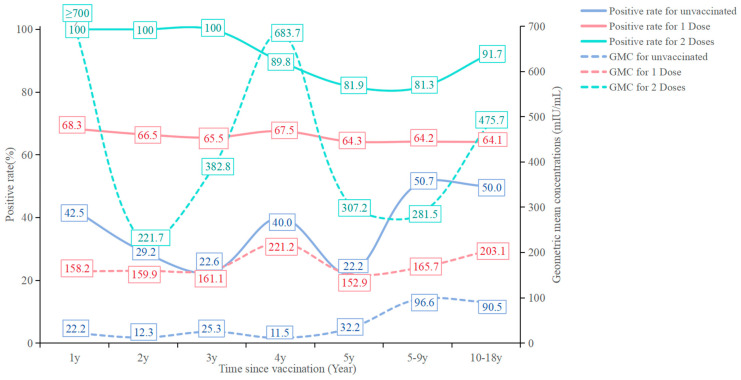
Positive rate and Geometric mean concentrations of varicella antibody for different vaccination status among subjects aged 0–18 years.

When analyzing across different vaccination statuses, no clear trend of increasing GMCs and positive rates with age was observed. Individuals who received one or two doses of the varicella vaccine had higher GMCs and positive rates of varicella antibodies compared to those who were unvaccinated. Moreover, antibody GMCs and positive rates increased with the number of vaccine doses administered. However, unlike those who received one or two doses, an increasing trend with age was noted in the unvaccinated group (Figure 1).

**Figure 2:**
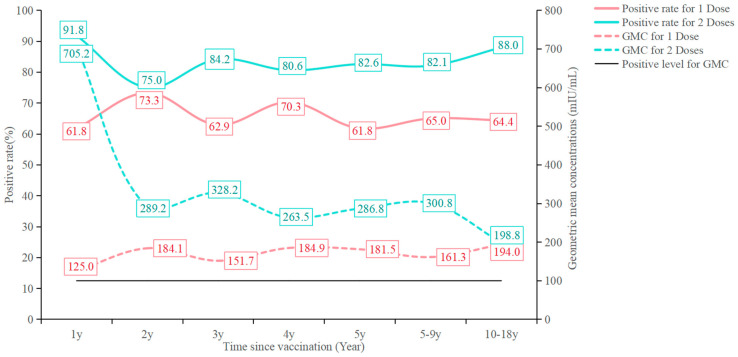
Positive rate and Geometric mean concentrations of varicella antibody for 1 or 2 doses among groups of time since vaccination.

Antibody titers diminish over time following vaccination, but they remain above the positive threshold (≥100 mIU/mL) even 6 years post-vaccination. For subjects who received two doses of the varicella vaccine, antibody levels fell from 705.2 mIU/mL to 198.8 mIU/mL, and positive rates dropped from 91.8% to 88.0% at 6 years post-vaccination. For those who received one dose, the antibody level fluctuates between 125.0 mIU/mL and 194.0 and positive rate fluctuates between 61.8% and 73.3% at 6 years post-vaccination (Figure 2).

In the multivariable regression analyses adjusted with a stepwise method, several factors were significantly associated with varicella IgG-specific antibody levels. These factors included area, sex, household registration, dose of varicella vaccine, and history of varicella (Table 2).

## 4. Discussion

Varicella antibodies among different age groups or after vaccination for different doses were analyzed in this study, which indicated that varicella antibody levels decreased as people became older but increased with the number of vaccines they received. The VZV antibody concentration were significantly associated with area, sex, household register, dose of varicella vaccine, and history of varicella.

Varicella antibody levels varied place to place all over the world. Compare with this study, Zhejiang 2015 [15] and Jiangxi 2017 [16]’s studies issued a similar varicella positive rate (62.99%, 63,27%). Liaonin 2020 [17] and Sichuan 2022 [18] have the lower positive rate (48.94%, 40.80%), while United States 2009–2010 [19] reported a much higher seroprevalence (across all age groups, from 97.1% to 98.9%). The different seroprevalence probably because of different varicella vaccination strategy and levels of varicella morbidity. The high varicella seroprevalence across all age in the US could be attributed to the successful implementation of routine varicella vaccination in the US starting in 1996. By 2008, one-dose varicella vaccination coverage among children aged 19–35 months reached 91% [19].

In concordance with Beijing 2017 and Liaonin 2020 [17], the seropositivity trend significantly increased with age, from 56.2% for 0 y to 98.6% to 40–49 y. A comparable study conducted in less developed areas of Guangdong, China, showed that the seropositivity rate increased gradually with age, reaching over 90% in the 20-30-year-old group [13]. This is mainly because of natural infection with VZV and implementation of varicella vaccine immunization. In our study, an increasing trend in antibody levels with age was observed in the unvaccinated group and the varicella antibody positive rates for subjects who had a history of varicella was much higher than those did not (92.2% vs. 56.7%). The same was observed in our cohort; seroprevalence decreased from subjects born in the natural infectious stage (before 1995) to subjects born in the promotion of the vaccination stage (after 1999) (92.1% vs. 63.8%). These investigations were conducted in settings where the wild-type varicella-zoster virus (VZV) was still circulating.

Our results indicated that females exhibited higher levels of antibodies. To elucidate the potential reasons, we conducted an analysis of the differences in history of varicella immunization and varicella disease between genders. No significant difference was found in the history of varicella vaccination between in men and women. It is noteworthy that the attack rates of varicella for females were approximately twice those of males (3.7% vs. 1.7%, *p* = 0.001, Supplementary Materials Table S1). However, a varicella epidemiology study in Guangzhou reached the opposite conclusion, showing that the varicella morbidity for males was higher than for females from 2005 to 2017, 143.9/100,000 per year vs. 116.6/100,000 per year [20]. To determine whether sampling bias exists, further studies are required to elucidate this intriguing paradox.

Vaccination help to improve the immunity level. In our recent study, we observed a correlation between the level of antibodies and the number of vaccine doses an individual has received. The data suggest that as the number of vaccine doses increases, so do the levels of antibodies in the vaccinated individuals. Subjects who received two doses of the vaccine had a higher GMC and positive rate than those who received one dose at any time since vaccination, and at any age. Our findings underscore the importance of potential for booster shots to elevate and maintain protective varicella antibody levels within the population.

Although antibody titers may diminish over time following vaccination, individual who received one or two doses of vaccines remain above the positive threshold (≥100 mIU/mL) even 6 years post-vaccination. The long-term maintenance of antibodies in healthy individuals after varicella vaccination has also been corroborated in a 14-year prospective study that evaluated the vaccine’s efficacy [21,22]. These findings highlight that while antibody levels may wane, they can still provide a level of protection against infection, even years after the administration of the vaccine. This is particularly relevant for public health strategies and vaccination programs, as it suggests that the positive effects of vaccination, in terms of antibody response, can be long lasting.

The varicella antibody levels were significantly higher in suburban and outer suburban areas compared to the central urban area. This disparity was found to be partially associated with the underlying vaccination rates across these regions, rather than the history of varicella infection (*p* > 0.05). The proportion of varicella vaccine uptake was notably lower in the central urban area as compared to the other two areas, with rates of 67.3% in the central urban area versus 70.7% in the suburban area and 69.5% in the outer suburban area (*p* = 0.001, see Supplementary Tables S2 for detailed data). The lower varicella vaccine uptake in central urban areas is a complex issue that involves multiple factors. The higher population density in urban areas may lead to relatively scarce medical resources and affects the convenience of vaccination services. In contrast, the lower population density in suburban and outer suburban areas results in a more balanced distribution of medical resources, making vaccination services more accessible. Additionally, the higher vaccination rate in suburban areas may be due to more vigorous promotion of vaccination programs. More efforts are required to reduce this disparity.

According to the World Health Organization’s position paper [2], a single-dose vaccine has a median effectiveness of 83% against varicella. While one dose is sufficient to reduce mortality and severe morbidity from varicella, it does not fully prevent limited virus circulation and outbreaks [2]. The two-dose regimen significantly decreases the number of cases and outbreaks, offering superior protection with a high effectiveness of 95% against all grades of severity of varicella [2]. It is hence recommended that both children and adults receive two doses of the varicella vaccine [23]. Considering the high incidence of varicella among children and in school settings, which poses a threat to children’s health and disrupts the educational continuity and learning environment, it is recommended that the varicella vaccine be integrated into the national routine immunization program. Extensive clinical validation and scientific studies over three decades have substantiated that routine varicella vaccination in children significantly reduces the incidence, hospitalization, and mortality rates associated with varicella [24,25,26,27]. Notably, a substantial reduction in disease burden has been observed across all age groups, including adults and infants who are not eligible for vaccination [28,29]. Recently, an increasing number of provinces and cities in China, such as Qingdao, Tianjin, Suzhou, Shanghai, Beijing, and Shenzhen, have incorporated varicella vaccination into their local immunization programs, achieving high vaccination rates [30].

We recognize that the demographic composition of our study sample introduces certain limitations in the generalizability of our findings. The primary objective of this monitoring is to assess the efficacy of the vaccine. Given that the majority of vaccinations and vaccine-preventable diseases occur among children and adolescents, our sampling population is predominantly composed of individuals within infants, children, and adolescents. It leads to a notable underrepresentation of adults and the elderly. Specifically, individuals within the age range of 10–59 years and those over 60 years account for only 17.7% and 4.5% of our sample, respectively. This distribution significantly deviates from the actual demographic composition in Guangzhou, which may undermine the reliability of our study’s outcomes when applied to adult and elderly populations. Consequently, the results obtained for these age groups may not be reproducible, and our conclusions should not be extrapolated indiscriminately to older cohorts. To address this, it is essential to refine the design of immunity level surveillance by sampling according to the actual demographic composition in Guangzhou, thereby ensuring a more representative and robust dataset that can accurately reflect the immunity levels across different age demographics.

Secondly, due to the developmental timeline of our information system and the inherent risk of recall bias, we did not collect vaccination records for a subset of subjects over 18 years old. Adults who are too old to have their records in the system and migrant children who have not been vaccinated in Guangzhou are likely not to have their information registered in the system, their vaccination files from other cities would not be accessible. This absence of records may be more prevalent among those who are unvaccinated, potentially overvaluing the VZV immunity level among the subjects. To ensure the accuracy of vaccination records, we obtained the vaccination records of the study subjects through information system, rather than relying on their personal recall, to avoid recall bias as possible.

Thirdly, the reliance on subjects’ memory for the collection of varicella history could introduce recall bias. Individuals who have been infected with VZV may be more inclined to accurately recall a history of varicella, while those without the disease may provide more ambiguous responses. This differential recall could lead to an overestimation of the associations between exposures and diseases, further complicating the interpretation of our findings.

## 5. Conclusions

The administration of two doses of varicella vaccine is recommended for both children and adults to achieve optimal protection against varicella. The integration of the varicella vaccine into the national routine immunization program is advocated in Guangzhou, China.

## Figures and Tables

**Table 1:** The geometric mean concentrations and positivity rate of varicella antibody under different categories for healthy population in 2020–2022.

Group	No. (%)	GMC (mIU/mL, 95% CI)	Positivity Rate (≥100 mIU/mL,%, 95% CI)
Age			
0 y	511 (15.5)	56.2 (45.1, 70.1)	42.5 (38.1, 46.9)
1 y	276 (8.4)	70.1 (53.8, 91.3)	51.1 (45, 57.1)
2 y	288 (8.7)	116.4 (90.8, 149.4)	61.1 (55.2, 66.8)
3 y	284 (8.6)	137.2 (108.1, 174)	63.7 (57.8, 69.3)
4 y	252 (7.6)	286.5 (222.1, 369.5)	73.8(67.9, 79.1)
5 y	211 (6.4)	216.8 (171.1, 274.6)	73.5 (67, 79.3)
5–9 y	745 (22.6)	218.6 (191.5, 249.5)	73 (69.7, 76.2)
10–19 y	204 (6.2)	228.1 (173.1, 300.4)	70.1 (63.3, 76.3)
20–29 y	195 (5.9)	361.4(262.4, 497.9)	78.5 (72, 84)
30–39 y	111 (3.4)	558.4 (406.7, 766.6)	90.1 (83, 94.9)
40–49 y	74 (2.2)	668.9 (505, 885.9)	98.6 (92.7, 100)
50-y	149 (4.5)	610.5 (472.3, 789.3)	95.3 (90.6, 98.1)
Doses			
0	779 (30.8)	46.5 (38.6, 56)	40.7 (36.4, 45.2)
1	1059 (41.9)	168.4 (151.4, 187.4)	65.3 (62.2, 68.2)
2	691 (27.3)	342.2 (305.8, 382.9)	83.4 (80.4, 86.1)
Sex			
Male	1672 (50.6)	143.5 (129.1, 159.5)	64.1 (61.7, 66.4)
Female	1628 (49.3)	205.2 (185, 227.7)	70 (67.7, 72.2)
Women of childbearing age (15–49 y)			
15–49 y	272 (16.7)	443.2 (349.5, 562)	83.1 (78.1, 87.3)
0–14 y and ≥50	1356 (83.3)	175.8 (156.9, 197)	67.4 (64.8, 69.9)
Household registration			
Local	2400 (72.7)	204.3 (187.9, 222)	70.8 (69, 72.6)
Nonlocal	646 (19.6)	110.6 (91.9, 133.1)	57.3 (53.4, 61.1)
Area			
Central urban	1201 (36.4)	120.8 (107.1, 136.2)	63.2 (60.4, 65.9)
Suburban	899 (27.2)	215.2 (185.9, 249.2)	68.9 (65.7, 71.9)
Outer suburban	1200 (36.4)	204.5 (181.1, 230.9)	69.4 (66.7, 72)
History of varicella			
0	2751 (83.4)	167.2 (154.3, 181.2)	66.7 (64.9, 68.4)
1	77 (2.3)	935.3 (676.8, 1292.4)	92.2 (83.8, 97.1)
Birth cohort			
-1994	368 (11.2)	579.1 (488.6, 686.3)	92.1 (88.9, 94.7)
1995–1998	77 (2.3)	300.1 (181, 497.4)	75.3 (64.2, 84.4)
1999–2012.9	384 (11.6)	241 (196.7, 295.3)	71.6 (66.8, 76.1)
2012.10–2017	973 (29.5)	210.8 (187.2, 237.4)	71.6 (68.7, 74.4)
2018-	1430 (43.3)	100 (88.6, 112.8)	56.4 (53.7, 59)
Year			
2020	1099 (33.30)	172.1 (150, 197.6)	63.4 (60.5, 66.3)
2021	1100 (33.33)	195 (172.7, 220.1)	67.8 (65, 70.6)
2022	1101 (33.36)	149.6 (131.8, 169.8)	69.8 (66.9, 72.5)

Abbreviations: No. (%), number (percent); CI, confidence interval; GMC, geometric mean concentration.

**Table 2:** Epidemiologic correlates the varicella IgG-specific antibody levels for healthy population in 2020–2022.

	Univariable Models			Multivariable Models		
Group	Estimate	Std. Error	*p*	Estimate	Std. Error	*p*
Age	0.015	0.001	<0.001	-	-	-
Sex	-	-	-	−0.117	0.03715	<0.05
Female	Ref.			-	-	-
Male	−0.145	0.033	<0.001	-	-	-
Household register	-	-	-	0.063	0.044	>0.05
Local	Ref.					
Nonlocal	0.172	0.040	<0.001			
Area				0.153	0.023	<0.001
Central urban	Ref.			-	-	-
Suburb	0.262	0.042	<0.001	-	-	-
Outer suburb	0.240	0.039	<0.001	-	-	-
Year						
2020	Ref.			-	-	-
2021	0.055	0.041	>0.05	-	-	-
2022	−0.058	0.041	>0.05	-	-	-
Year group				-	-	-
1	Ref.			-	-	-
2	−0.257	0.115	<0.05	-	-	-
3	−0.381	0.067	<0.001	-	-	-
4	−0.439	0.056	<0.001	-	-	-
5	−0.768	0.054	<0.001			
Doses				0.440	0.025	<0.001
0	Ref.			-	-	-
1	0.566	0.042	<0.001	-	-	-
2	0.874	0.046	<0.001	-	-	-
History of varicella				0.697	0.169	<0.001
No	Ref.			-	-	-
Yes	0.695	0.108	<0.001	-	-	-

## Data Availability

The data presented in this study are available on request from the corresponding author due to legal reason.

## Supplementary Materials

The following supporting information can be downloaded at: https://www.mdpi.com/article/10.3390/vaccines13010023/s1, Table S1: The subjects’ vaccination dosage and history of varicella by sex; Table S2: The subjects’ vaccination dosage and history of varicella by area.
