# Supplementary material for: Optimizing Varicella Vaccination Strategy: A Study on Age and Dose Impacts on Antibody Levels
Source: Vaccines (Basel). 2024 Dec 30;13(1):23. doi: 10.3390/vaccines13010023 (PMC11769036; doi:10.3390/vaccines13010023)
Supplement: Supplementary file 1 [file vaccines-13-00023-s001.zip › vaccines-3319158-supplementary.pdf]

Table S1. The subjects’ vaccination dosage and history of varicella by sex.

Doses	Vaccination dosage(%)			Ever infected with varicella (%)	
	0	1	2	No	Yes
Female	351(29.9)	495(42.2)	328(27.9)	1377(96.3)	53(3.7)
Male	439(31.8)	574(41.6)	367(26.6)	1374(98.3)	24(1.7)
Total	790(30.9)	1069(41.9)	695(27.2)	2751(97.3)	77(2.7)

Table S2. The subjects’ vaccination dosage and history of varicella by area.

Area	Vaccination dosage(%)			Ever infected with varicella (%)	
	0	1	2	No	Yes
Central urban	291(32.7)	335(37.6)	265(29.7)	1047(96.7)	26(2.4)
Suburban	208(29.3)	292(41.1)	210(29.6)	831(96.3)	32(3.7)
Outer suburban	291(30.5)	442(46.4)	220(23.1)	873(97.9)	19(2.1)
Total	790(30.9)	1069(41.9)	695(27.2)	2751(97.3)	77(2.7)
